# Effect of dynamic balance on human mental rotation task in female badminton vs. volleyball players

**DOI:** 10.3389/fpsyg.2023.1338265

**Published:** 2024-01-11

**Authors:** Samiha Amara, Badria Al-Hadabi, Heba El-Ashkar, Nabil Gmada, Hamdi Habacha, Bessem Mkaouer

**Affiliations:** ^1^Physical Education and Sport Sciences Department, College of Education, Sultan Qaboos University, Muscat, Oman; ^2^Department of Individual Sports, Higher Institute of Sport and Physical Education of Ksar Said, Manouba University, Tunis, Tunisia; ^3^Department of Water Sports Training, Faculty of Physical Education for Girls, Helwan University, Helwan, Egypt; ^4^Department of Biological Sciences, Higher Institute of Sport and Physical Education of Kef, University of Jendouba, El Kef, Tunisia; ^5^Integrative Neuroscience and Cognition Center, Université de Paris, CNRS, Paris, France

**Keywords:** mental rotation, response time, dynamic balance, badminton, volleyball

## Abstract

**Background:**

The present study aims to compare the mental rotation performance between two non-contact sports (i.e., badminton and volleyball) in different upright conditions (i.e., with and without dynamic balance).

**Methods:**

Thirty-five female sports and physical education students voluntarily participated in the experiment, including fourteen specialists in badminton and twenty-one specialists in volleyball. The experiment involved a mental body rotation task with or without balance exercises on a wobble board.

**Results:**

Badminton players outperformed volleyball players in the mental rotation tasks regardless of balance. More interestingly, the results revealed an overall decrease in reaction times when participants performed balance exercises simultaneously with mental rotation.

**Discussion:**

Our findings suggest that introducing dynamic balance on a wobble board has immediate beneficial effects on the mental rotation performance of female badminton and volleyball players. These findings are discussed in the context of sport specificities and cognitive processing framework.

## 1 Introduction

Brain imaging studies provide strong evidence for the involvement of the body’s mirror system in observing complex movements (Calvo-Merino et al., 2005). Guillot and Collet (2005) showed that imagining a movement seems to preserve the spatial and temporal characteristics and be based on the same cognitive and neural systems as the actual movement. In a mental rotation task (MR), participants are asked to identify as fast as possible whether two misoriented images represent the same or mirrored object. Such object-based mental rotation tasks classically tap on visual processes, implying a linear increase in decision times as a function of the angular disparity between the two images (Shepard and Metzler, 1971). Later, some authors used images of body parts (i.e., hand or foot) in MR tasks and asked participants to judge whether the image depicted a right or left body part (i.e., laterality judgment). The results revealed that reaction times were affected by the biomechanical constraints of the real body parts movements (Sekiyama, 1982). These findings suggest that participants imagine their own body parts moving until alignment with the position of the stimulus (Moreau, 2012, 2013). Similarly, studies using depictions of human bodies with one arm outstretched revealed that the time to judge which arm is outstretched (i.e., laterality judgment) is dramatically affected by extreme (i.e., upside-down) body positions (Steggemann et al., 2011). Hence, these egocentric mental rotation tasks seem to imply embodied motor strategies transforming participants’ own mental body representations to solve the task (Steggemann et al., 2011; Habacha et al., 2022; Khalfallah et al., 2022). That is, egocentric mental rotation involves cognitive processes used for both motor imagery and motor execution (Kawasaki and Higuchi, 2013).

Sport practice is an ideal context to develop spatial capacities, in particular visualization, orientation, and MR (Calvo-Merino et al., 2005; Pietsch and Jansen, 2012). During sports practice, the cognitive mechanisms underlying a movement play a key role in its improvement, considering the functional equivalence between actual and imagined skills (Decety, 2002). Moreau et al. (2012) and Pietsch and Jansen (2012) demonstrated large effects of motor training on mental rotation performance.

Additionally, athletes with different abilities in different sports appear to use different strategies to solve the same mental rotation tasks. Accordingly, the specific sensorimotor experiences seem to shape the cognitive processing during these tasks (Steggemann et al., 2011; Habacha et al., 2017, 2022). These findings support the involvement of motor processes in MR (Jansen and Lehmann, 2013) and further refine the established equivalence between actual and covert movement.

Furthermore, it has been shown that physical activity, especially a balance training program, improves memory and spatial awareness (Rogge et al., 2017). Rogge et al. (2017) compared the balance and cardiorespiratory fitness of two groups with and without 12 weeks of balance training. Only participants who followed the training significantly improved their balance, memory, and spatial awareness. The researchers explain that stimulation of the vestibular system during balance training could have induced changes in the hippocampus and parietal cortex, possibly through direct pathways between the vestibular system and these brain regions (Rogge et al., 2017). Bigelow and Agrawal (2015) showed a link between vestibular function and cognitive domains of visuospatial ability, including spatial memory, navigation, MR, and mental representation of three-dimensional space. Hofmann and Jansen (2021) investigated the relationship between MR and postural stability by examining the effects of performing an egocentric (i.e., bodily stimuli) and object-based (i.e., abstract stimuli) MR task simultaneously with stabilized postural sway in a tense position with both legs on a stable surface (i.e., a force plate). Their results showed that the egocentric task involved more body swaying than the object-based task. These results suggest that the egocentric mental rotation task involved more kinaesthetic imagery and motor processes in that subjects had to imagine rotating their own bodies’ mental representations (Kessler and Rutherford, 2010), whereas the object-based task involves mostly visual processes that are not affected by the kinaesthetic body representations (Hofmann and Jansen, 2021). Furthermore, increasing the rotation angle of the stimuli in the MR task resulted in more body sway (Hofmann et al., 2022), confirming the involvement of motor processes. Pellecchia (2003) corroborated this finding by revealing that more body sway may be due to increased difficulty of concurrent cognitive tasks. In addition, attentional focus (i.e., internal and external) is very important. Mental body rotation combined with dynamic balance engages both external and internal attentional focus, noting that the performance benefits are greatest when participants use an external focus of attention (e.g., directed attention on the effect of movement on the environment) versus an internal focus of attention (e.g., a focus on body movement) (Wulf et al., 1999, 2010; Singh and Wulf, 2022).

However, not all athletes automatically engage motor processes during MR of bodily stimuli, resulting in contradictory results. Participation in certain sports, such as wrestling, seems to favor motor-based strategies to outperform other athletes or non-athletes, even if abstract objects are used in MR (Moreau et al., 2012; Pietsch et al., 2019). Furthermore, athletes whose sports require more visuospatial and kinaesthetic abilities linked to real body rotations, such as wrestlers and gymnasts, show better performance in MR of bodily stimuli than athletes who practice cardiovascular activities such as running (Moreau et al., 2012; Schmidt et al., 2016). In contrast, team sports encourage the use of visual strategies, as athletes are trained to perceive and analyze moving objects and examine spatial relationships with partners and opponents from off-centre perspectives (Steggemann et al., 2011).

Moreover, athletes from team sports and racquet sports showed significantly shorter reaction times (i.e., go/no-go) than those in other sports (Dogan, 2009; Erickson, 2020). Delpont et al. (1991) observed faster transmission in the visual pathway in tennis and squash players compared to rowers and non-athlete controls. That is, team sports and racquet sports, which require rapid visual activity, seem to enhance the development of information processing and mental rotational performance. However, other studies have shown that elite team athletes do not exhibit better mental rotational performance of bodily and abstract figures compared to non-athletes (Jansen et al., 2012; Heppe et al., 2016).

One way to provide new insight into these contradictory results is to compare the performance of badminton and volleyball female players in MR of bodily stimuli in different upright conditions (i.e., with and without dynamic balance). The effect of balance on MR performance in one group and not the other would help understand the processes engaged in the task.

We hypothesized that the dynamic balance condition would have immediate beneficial effects on the MR task, resulting in decreased response times for both female badminton and volleyball players. Additionally, we made the hypothesis that female badminton players would be more effectively able and faster than their volleyball counterparts to recognize the correct response of rotated body images, given that the shuttlecock in badminton travels at a much faster and less predictable trajectory than the ball in volleyball.

## 2 Methods

### 2.1 Participants

A minimum sample size of 35 participants was determined from an *a priori* statistical power analysis using G*Power software [Version 3.1, University of Dusseldorf, Germany (Faul et al., 2009)]. The power analysis was computed with an assumed power of 0.95 at an alpha level of 0.05 and a moderate effect size of 0.30. Therefore, thirty-five volunteer female sports and physical education students, fourteen specialists in badminton (age 20.48 ± 1.04 years; height 1.80 ± 0.03 m; weight 78.12 ± 3.73 kg) and twenty-one specialists in volleyball (age 21.57 ± 1.47 years; height 1.87 ± 0.02 m; weight 80.03 ± 4.03 kg) agreed to participate in this study. After being informed in advance of the procedures, methods, benefits, and possible risks of the study, each participant reviewed and signed a consent form to participate in the study. The experimental protocol was performed in accordance with the Declaration of Helsinki for human experimentation (Carlson et al., 2004) and was approved by the University Local Ethical Committee (EDU/PHEDS83961/2022).

### 2.2 Experimental design and procedures

This study consists of three random assessments (i.e., randomized counterbalanced, Latin Square), every assessment took place on a separate successive day. All assessments were carried out in the gymnasium at the same time of the day (between 10:00 PM and 12:00 PM). Each of the assessments was a human mental rotation task with and/or without balance exercises, i.e., (frontal and/or sagittal balance) on a wobble board SPBB [length and width 420 mm × 420 mm; height 70 mm (Mattacola and Lloyd, 1997)].

**Figure 1:**
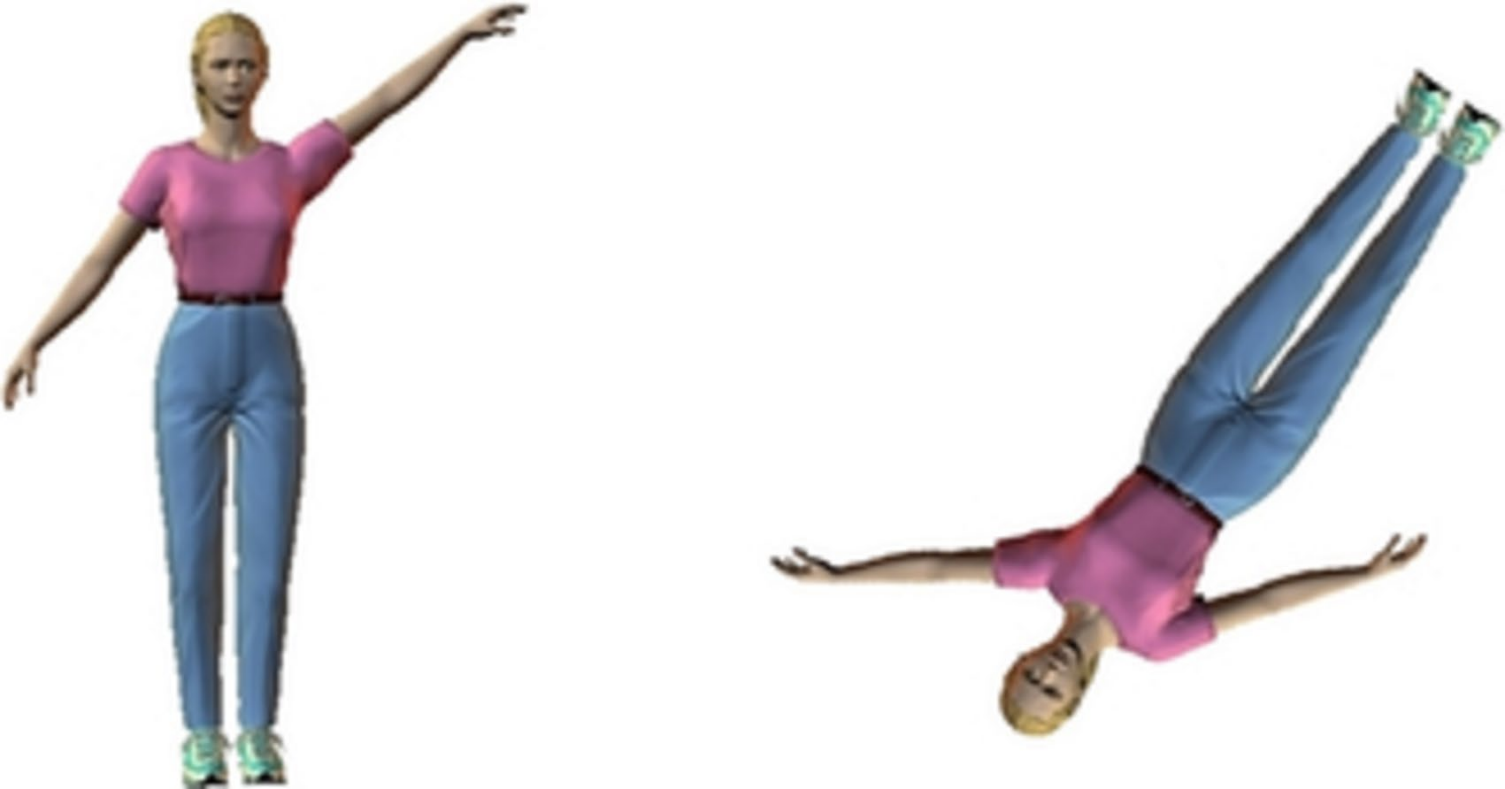
Example of stimulus of objected-based body condition.

Five stimuli were used in the mental rotation task with egocentric transformation (ET), each including a pair of standard and comparison images (Figure 1). We used the standard image on the left part of the monitor screen and the rotated image on the right of the screen. The standard image consists of an upright human figure with either the left or right arm outstretched. The comparison image was either a copy or a mirror-reversed copy of the standard one, rotated in one of five orientations (45°, 135°, 180°, 225°, and 315°).

**Figure 2:**
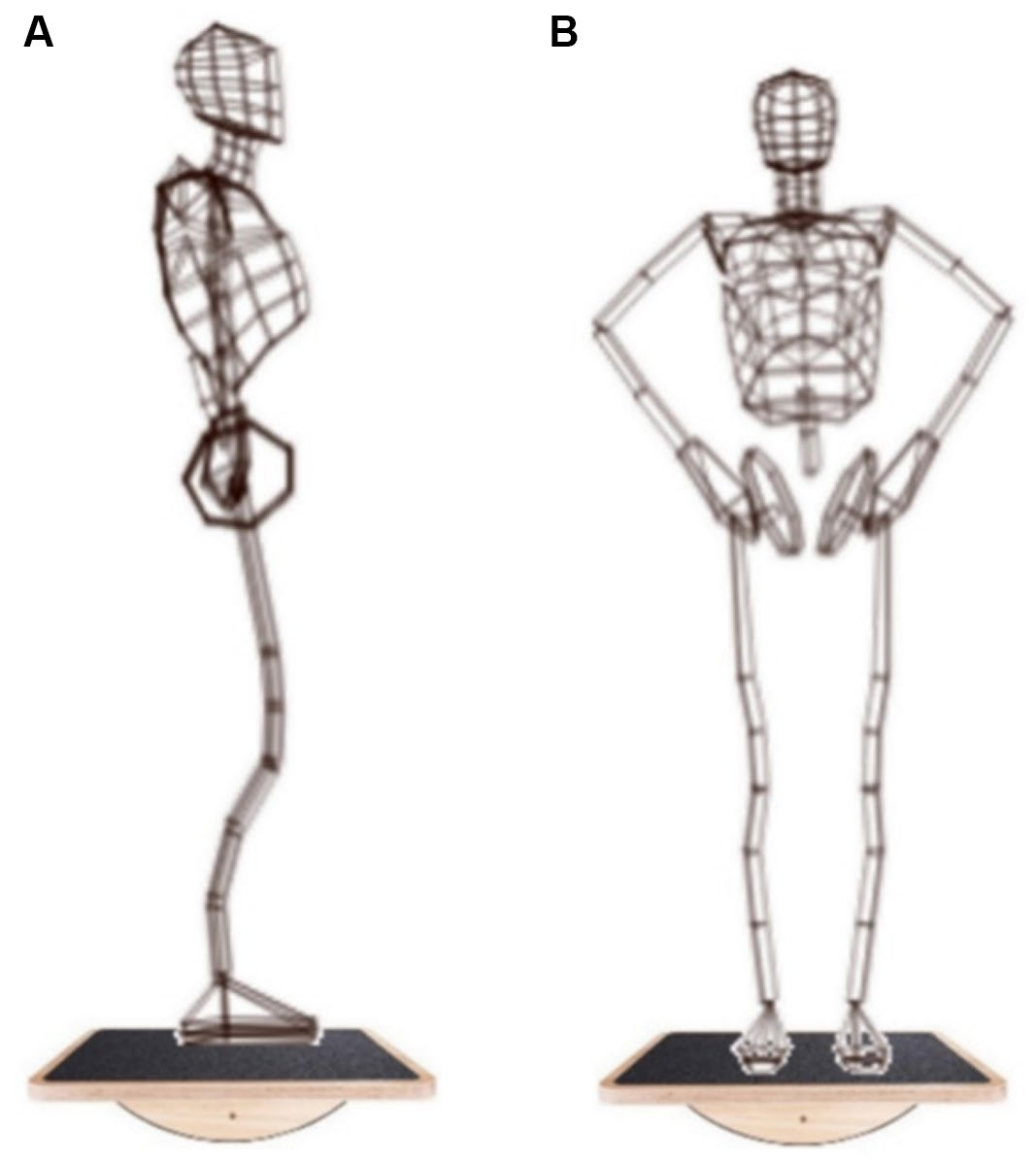
Experimental protocol: **(A)** Bipedal sway, sagittal balance; **(B)** Bipedal sway, frontal balance (El-Ashkar et al., 2023).

The mental rotation task was performed in three conditions:

- In a standing position: The subject takes an upright position in front of the PC with a wireless joystick in his hand.
- In sagittal balance: The subject takes an upright position on a Single Plane Balance Board (SPBB) in front of the PC with a wireless joystick in his hand (Figure 2A).
- In frontal balance: The subject takes an upright position on a Single Plane Balance Board (SPBB) in front of the PC with a wireless joystick in his hand (Figure 2B).

This results in a total of 60 trials: 3 (conditions: static, sagittal and frontal balance) × 2 (groups: badminton and volleyball) × 5 (angle display: 45°, 135°, 180°, 225°, and 315°) × 2 (same or different). The order of stimulus presentation was counterbalanced, and each rotation angle could not appear 2 times in succession.

Each trial began with a blank screen for 1,000 ms, after which a black fixation cross was displayed at the centre for 500 ms. After fixation, the test image was presented for a maximum of 5,000 ms and remained on the screen until a response was given. Stimuli were displayed, and response times were recorded via the free software OpenSesame (Mathôt et al., 2012). The mental rotation task lasted about 4 min.

### 2.3 Statistical analysis

The SPSS 20 package [SPSS, Chicago, IL, USA] program was used for the data analysis. Descriptive statistics (i.e., means ± SD) were performed for all variables. The effect size was conducted using G*Power software [Version 3.1, University of Dusseldorf, Germany]. The following scale was used for the interpretation of *d*: <0.2, trivial; 0.2–0.6, small; 0.6–1.2, moderate; 1.2–2.0, large; and > 2.0, very large (Hopkins, 2002). The normality of distribution estimated by the Kolmogorov–Smirnov test was acceptable for all variables (*p* > 0.05). Consequently, ANOVA with repeated measures on two factors (i.e., balance and group) was used to benchmark different balance strategies. The Bonferroni test was applied in post-hoc analysis for pairwise comparisons. Additionally, effect sizes (*d*) were determined from ANOVA output by converting the partial eta-squared to Cohen’s *d*. *A priori* level less than or equal to 0.5% (*p* ≤ 0.05) was used as a criterion for significance.

## 3 Results

The ANOVA showed a significant main effect of “balance” (i.e., without balance (WB), with sagittal (SB) and frontal balance (FB)) and “group” (i.e., badminton and volleyball) in the response time (*p* < 0.01) and the error percentage (*p* < 0.05). In addition, results revealed a significant interaction between “balance” and “group” [in general RT (Table 1)].

**Table 1:** ANOVA with repeated measures.

Source		df	Mean square	*F*	Sig.	Effect size	Power
Balance	RT Gen	2	3175776.700	15.115	0.000**	1.754	0.999
	EP Gen	2	47.657	1.180	0.314	0.380	0.250
	RT 45°	2	1711623.410	7.018	0.002**	1.353	0.917
	EP 45°	2	124.813	2.548	0.086	0.557	0.493
	RT 90°	2	2541526.356	8.211	0.001**	1.235	0.953
	EP 90°	2	21.084	0.239	0.788	0.167	0.086
	RT 135°	2	4090458.058	13.037	0.000**	1.256	0.996
	EP 135°	2	503.038	2.107	0.130	0.505	0.418
	RT 180°	2	5474120.555	12.349	0.000**	1.222	0.995
	EP 180°	2	324.304	1.093	0.341	0.363	0.234
	RT 225°	2	6258626.916	10.216	0.000**	1.111	0.983
	EP 225°	2	92.726	0.490	0.615	0.246	0.127
	RT 270°	2	1746830.048	4.253	0.018*	0.717	0.725
	EP 270°	2	71.822	0.674	0.513	0.285	0.159
	RT 315°	2	9182409.417	32.906	0.000**	1.996	1.000
	EP 315°	2	710.680	4.420	0.016*	0.731	0.742
Sports	RT Gen	1	1495019.892	4.170	0.048*	0.716	0.539
	EP Gen	1	1842.046	3.924	0.050*	0.698	0.503
	RT 45°	1	1625891.593	2.829	0.102	0.585	0.372
	EP 45°	1	540.043	1.522	0.226	0.429	0.224
	RT 90°	1	649695.123	1.120	0.298	0.369	0.177
	EP 90°	1	754.204	1.787	0.190	0.463	0.255
	RT 135°	1	20097.730	0.027	0.870	0.063	0.053
	EP 135°	1	2546.911	3.549	0.068	0.655	0.448
	RT 180°	1	72024.306	0.055	0.815	0.089	0.056
	EP 180°	1	5070.882	5.458	0.026*	0.813	0.621
	RT 225°	1	386098.537	0.251	0.620	0.179	0.078
	EP 225°	1	1477.890	1.702	0.201	0.454	0.245
	RT 270°	1	1620162.190	2.150	0.152	0.509	0.296
	EP 270°	1	2415.585	4.628	0.039*	0.749	0.551
	RT 315°	1	142798.030	0.246	0.623	0.007	0.167
	EP 315°	1	1333.900	4.174	0.049*	0.695	0.509
Balance * Sports	RT Gen	2	121424.870	5.530	0.018*	0.715	0.677
	EP Gen	2	1.317	0.033	0.968	0.063	0.055
	RT 45°	2	248845.956	1.020	0.366	0.351	0.221
	EP 45°	2	13.676	0.279	0.757	0.179	0.092
	RT 90°	2	204010.169	0.659	0.521	0.285	0.156
	EP 90°	2	59.082	0.670	0.515	0.285	0.158
	RT 135°	2	236124.603	0.753	0.475	0.300	0.173
	EP 135°	2	124.774	0.523	0.595	0.255	0.133
	RT 180°	2	152109.886	0.343	0.711	0.201	0.103
	EP 180°	2	60.048	0.202	0.817	0.155	0.080
	RT 225°	2	50449.177	0.082	0.921	0.089	0.062
	EP 225°	2	77.672	0.410	0.665	0.220	0.114	
	RT 270°	2	81556.607	0.199	0.820	0.155	0.080
	EP 270°	2	34.887	0.328	0.722	0.201	0.100
	RT 315°	2	175709.665	0.630	0.536	0.278	0.151
	EP 315°	2	7.053	0.044	0.957	0.063	0.056

RT, Response time; EP, Error percentage; Gen, General; *Significant at *p* < 0.05; **Significant at *p* < 0.001.

**Figure 3:**
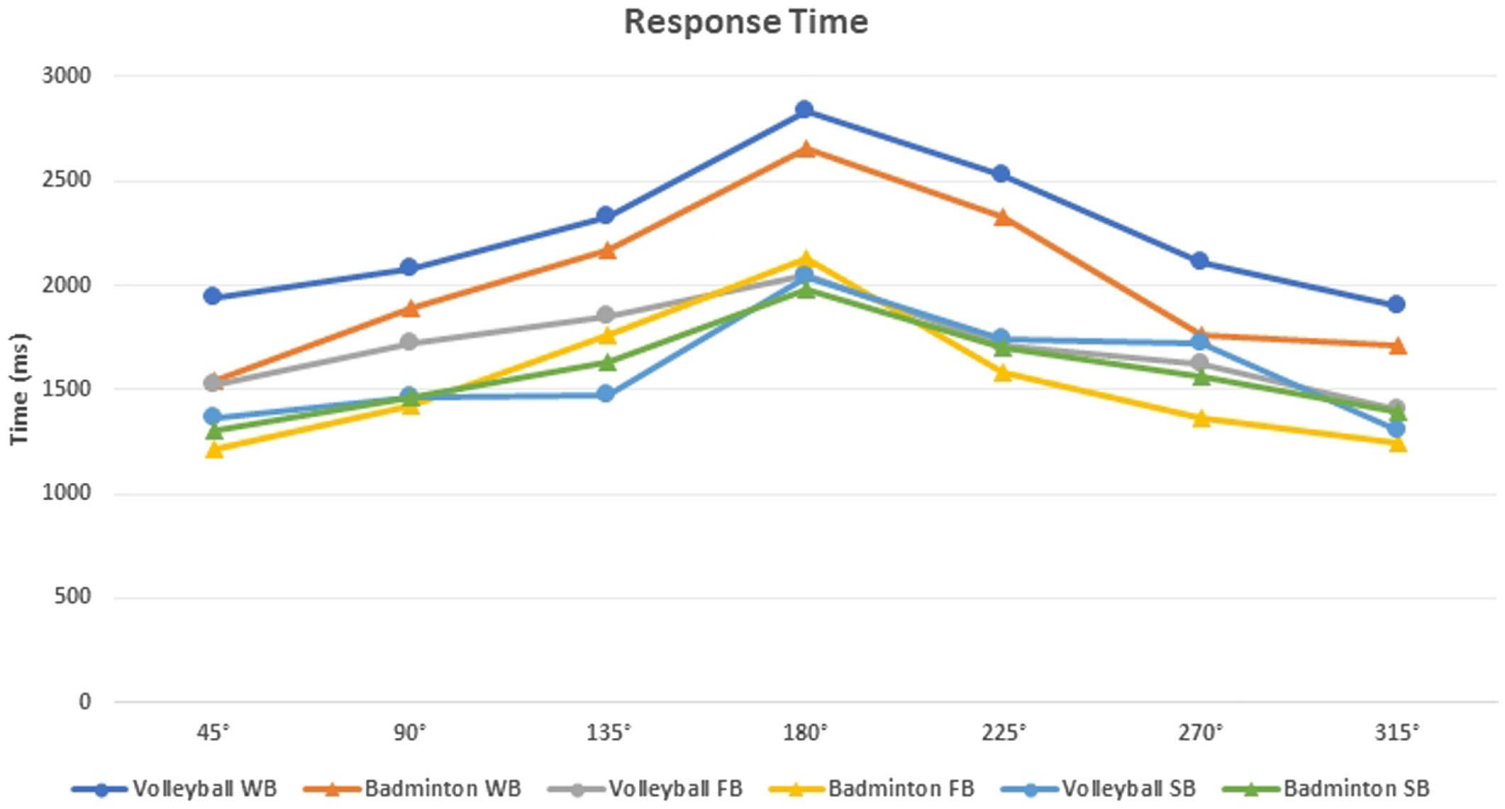
Response time for all body rotation angles and conditions.

Pairwise comparison between balance conditions (i.e., without balance, with sagittal and frontal balance) showed significant differences (*p* < 0.01) for RT in all rotation degrees (i.e., 45°, 90°, 135°, 180°, 225°, 270°, and 315°) between WB and FB conditions. Also, between WB and SB conditions. In addition, there is a significant RT difference (*p* < 0.01) between FB and SB only in 135° rotation. Then, the EP results showed a significant difference (*p* < 0.05) in 315° body rotation angle, in WB vs. FB conditions (6.19 ± 13.45% and 3.47 ± 8.15% respectively) and FB vs. SB conditions (3.47 ± 8.15% and 5.23 ± 9.71% respectively) (Figure 3; Table 2).

**Table 2:** Pairwise comparison.

Measure		Mean diff	Std. error	Sig.	Effect size
RT Gen	WB vs. FB	505.871	123.914	0.000**	4.082
	WB vs. SB	555.627	125.253	0.000**	4.346
	BB vs. AB	49.756	80.473	0.541	0.618
RT 45°	WB vs. FB	370.229	127.237	0.006**	2.910
	WB vs. SB	408.769	143.842	0.008**	2.841
	FB vs. SB	38.539	81.677	0.640	0.471
RT 90°	WB vs. FB	412.583	152.637	0.011*	2.703
	WB vs. SB	521.337	152.319	0.002**	3.421
	FB vs. SB	108.753	93.654	0.254	1.161
RT 135°	WB vs. FB	438.344	158.909	0.009**	2.758
	WB vs. SB	689.396	150.210	0.000**	4.589
	FB vs. SB	251.052	90.617	0.009**	2.770
RT 180°	WB vs. FB	655.749	172.691	0.001**	3.797
	WB vs. SB	735.619	166.801	0.000**	4.431
	FB vs. SB	79.870	146.669	0.590	0.541
RT 225°	WB vs. FB	784.572	242.246	0.003**	3.242
	WB vs. SB	703.960	197.011	0.001**	3.753
	FB vs. SB	80.612	109.077	0.465	0.733
RT 270°	WB vs. FB	448.896	152.259	0.006**	2.953
	WB vs. SB	293.990	150.947	0.060	1.953
	FB vs. SB	154.906	165.470	0.356	0.933
RT 315°	WB vs. FB	916.910	143.518	0.000**	6.411
	WB vs. SB	893.558	139.367	0.000**	6.428
	FB vs. SB	23.352	99.047	0.815	0.232
EP 315°	WB vs. FB	7.539	3.479	0.038*	2.513
	WB vs. SB	0.794	1.568	0.616	0.012
	FB vs. SB	8.333	3.762	0.034*	2.777

RT, Response time; EP, Error percentage; Gen, General; WB, without balance; FB, Frontal balance; SB, Sagittal balance; *Significant at *p* < 0.05; **Significant at *p* < 0.001.

Furthermore, between-group comparison (i.e., badminton vs. volleyball), showed a significant difference (*p* < 0.05) in the general RT (1644.91 ± 465.02 ms vs. 2182.08 ± 684.24 ms respectively) and general EP (2.89 ± 3.11% vs. 11.22 ± 16.63% respectively). Also, in the EP at 180°, 270° and 315° body rotation angle in WB (180° = 3.57 ± 7.10% vs. 19.05 ± 24.88%, 270° = 1.19 ± 4.46 vs. 12.70 ± 18.18% and 315° = 1.20 ± 4.44 vs. 9.52 ± 18.30% respectively), FB (180° = 7.17 ± 14.21% vs. 23.14 ± 29.51% and 270° = 2.38 ± 6.05 vs. 12.71 ± 18.41% respectively) and SB (315° = 1.18 ± 4.44 vs. 7.93 ± 11.32% respectively) conditions.

Regarding the balance * group interaction, there is a significant difference (*p* < 0.05) in the general RT between badminton and volleyball players, particularly in WB condition (1644.91 ± 465.02 ms vs. 2182.07 ± 24.09 ms respectively). When introducing the FB or SB task, the RTs are very close (1518.42 ± 405.40 ms vs. 1701.58 ± 554.86 ms and 1555.41 ± 372.47 ms vs. 1565.08 ± 433.56 ms respectively, badminton and volleyball players).

**Figure 4:**
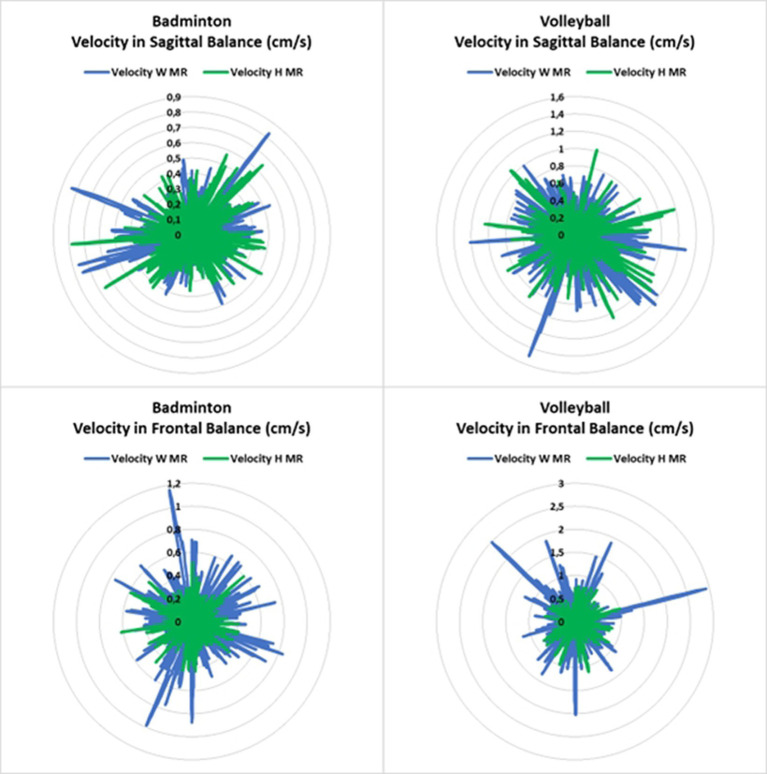
Balance velocity in frontal and sagittal conditions with and without human mental rotation task.

**Figure 5:**
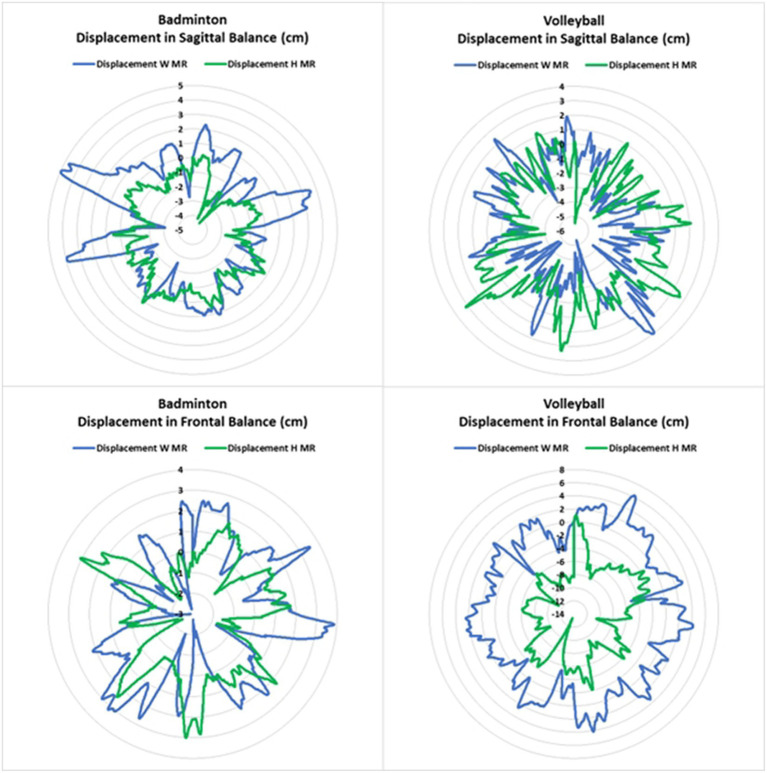
Balance displacement in frontal and sagittal conditions with and without human mental rotation task.

On the other side, balance (i.e., velocity and displacement) was enhanced when introducing MR task (*p* < 0.01) in both sports disciplines (i.e., volleyball and badminton) and balance conditions (i.e., FB and SB) (Figures 4, 5). In addition, there is a significant interaction between FB and sports in the displacement [*F*_(1,33)_ = 4.333; *p* < 0.05; *d* = 0.876] in favor of badminton players (10.886 ± 4.029 cm vs. 8.053 ± 3.172 cm, *p* < 0.001, *d* = 1.256, respectively volleyball and badminton players).

## 4 Discussion

This study aimed to compare the MR performance between two non-contact sports, namely badminton and volleyball, across different upright conditions (i.e., with and without dynamic balance) in female players. More specifically, the aim was to examine whether dynamic balance affect the performance of these female athletes implying their use of motor processes during the task.

Our results showed a significant decrease of RT in both balance conditions (FB and SB) compared to the static condition (WB). This finding suggests that the unstable equilibrium position could have enhanced the cognitive processing of participants allowing them to perform the MR task faster. In this context (Kawasaki et al., 2014), showed that participants in unipedal standing performed the MR task faster than the bipedal standing group and had lower sway scores. They revealed that the MR is involved in controlling upright human posture and could be related to the ability to stand as still as possible. Taken together with our results, these findings suggest that the ability to mentally imagine body movements may be related to postural stability while involving a challenging postural task. Thus, our results are consistent with those of Kawasaki and Higuchi (2013), which showed that MR interventions have immediate beneficial effects using dynamic balance conditions.

Additionally, the decrease of RT in balance conditions was significant in all body rotation angles, ranging from body orientations that every athlete could execute during practice to extreme body positions. That is, cognitive processing seems to be enhanced even with stimuli that require easy or little processing, highlighting the robustness of the effect of balance. Accordingly, our results confirm that a dual task (i.e., MR and body sway) enhances both performances (i.e., RT and stability) in FB and SB conditions (Hofmann and Jansen, 2021; Hofmann et al., 2022). In addition, Rogge et al. (2017) proved beneficial effects on memory, orientation, and spatial cognition after balance training through the activation of the vestibular system.

Furthermore, our results reveal a classic linear increase of RT as a function of rotation angle up to 180° and a decrease after. Habacha et al. (2022) and Steggemann et al. (2011) computed mean RT of angular disparities for which the shortest rotation path between stimulus and target is the same (e.g., 45°–315°, 90°–270°, 135°–225°). In addition, Parsons (1987) and Zartor et al. (2010) confirm that participants in mental body rotation tasks classically choose the shortest path to align their body representation with the stimuli. This interpretation can explain why the 180° angle represents the greatest difficulty in our study.

Keehner et al. (2006) and Michelon and Zacks (2006) revealed that the classical behavioral result for egocentric MR tasks implies an increase of RT for angles above 60° or 90°. Angles below 60° detect body positions that are similarly easy to physically and mentally adopt and thus show no increase in RT. In our study, the introduction of a second task (i.e., postural balance) could have made the easy positions difficult to adopt, since the balance affected the starting position of the body representation to transform. This is in line with the studies showing that the change in body position during MR can affect performance (Ionta et al., 2007; Ionta and Blanke, 2009). That is, more cognitive processing could have been required and thus RT increased even for small angles. Furthermore, Huxhold et al. (2006) and Shumway-Cook and Woollacott (2007) suggest that postural stability is the result of a shift of attention to the cognitive task and therefore the automaticity and efficiency of the postural control processes are enhanced. Against this, Hofmann and Jansen (2021) revealed that in egocentric tasks, the angular disparity does not influence postural sway. For object-based tasks, there is a tendency for higher rotation angles to lead to more postural sway.

Our results revealed that female badminton players have faster RT and smaller error percentages than female volleyball players. This could be explained by the fact that, in badminton, the shuttlecock travels at a much higher speed and with a less predictable trajectory than a volleyball, requiring faster reflexes to be able to hit the shuttlecock accurately (Phomsoupha and Laffaye, 2015). Additionally, female badminton players often must react to shots that are hit directly at them, whereas female volleyball players have more time to react because the ball is hit back and forward across a net. Moreover, research studies have shown that badminton players tend to have faster reaction times compared to other athletes practicing other sports such as football, handball, volleyball, wrestling, and ice skating (Bańkosz et al., 2013; Dube et al., 2015). Consequently, Alexander and Boreskie (1989) classified sports such as badminton, table tennis, and squash (i.e., with racquets) as reaction sports.

Interestingly, the interaction between balance and groups was only significant in the WB condition. When introducing FB or SB tasks, the RTs were fairly similar. This suggests that both female groups react in the same way to stress/disturbance of postural balance on both the frontal and sagittal planes (Bisht et al., 2017). Bisht et al. (2017) showed no significant difference in balance ability between volleyball and badminton players and suggested that balance ability is equally necessary and a prerequisite for both of these sports. That is, badminton and volleyball players could similarly develop their balance skills during their practice, and a disturbance in their balance would thus induce almost the same reaction (Trivedi and Rawal, 2020).

## 5 Conclusion

In summary, introducing dynamic balance on a wobble board seems to have benefits on the performance of a concurrent mental rotation task, with similar benefits observed in both female badminton and volleyball players. Additionally, the superior performance of female badminton players compared to volleyball players suggests distinct effects of these two sports on mental rotation abilities. Furthermore, dynamic balance seems to be equally necessary and a prerequisite for both female badminton and volleyball players.

## Data availability statement

The raw data supporting the conclusions of this article will be made available by the authors, without undue reservation.

## Ethics statement

The studies involving humans were approved by Sultan Qaboos Local Ethical Committee (EDU/PHEDS83961/2022). The studies were conducted in accordance with the local legislation and institutional requirements. The participants provided their written informed consent to participate in this study. Written informed consent was obtained from the individual(s) for the publication of any potentially identifiable images or data included in this article.

## Author contributions

SA: Conceptualization, Data curation, Funding acquisition, Methodology, Project administration, Software, Writing – original draft, Writing – review & editing. BA-H: Funding acquisition, Investigation, Project administration, Resources, Writing – review & editing. HE-A: Investigation, Methodology, Project administration, Writing – review & editing. NG: Project administration, Resources, Supervision, Visualization, Writing – review & editing. HH: Conceptualization, Methodology, Validation, Writing – review & editing. BM: Conceptualization, Data curation, Formal analysis, Methodology, Project administration, Software, Supervision, Writing – review & editing.
